# Sodium-Glucose Co-Transporter 2 Inhibitors Use Improves the Satisfaction With Anti-diabetic Agent Treatment: A Questionnaire-based Propensity Score-matched Study

**DOI:** 10.3389/fphar.2021.787704

**Published:** 2022-02-01

**Authors:** Fang-Hong Shi, Jiang Yue, Yi-Hong Jiang, Ming-Lan Yang, Zhi-Chun Gu, Jing Ma, Hao Li

**Affiliations:** ^1^ Department of Pharmacy, Ren Ji Hospital, Shanghai Jiao Tong University School of Medicine, Shanghai, China; ^2^ Department of Endocrinology, Ren Ji Hospital, Shanghai Jiao Tong University School of Medicine, Shanghai, China; ^3^ Department of Pharmacy, Clinical Research Center, Shanghai Children’s Medical Center, School of Medicine, Shanghai Jiao Tong University, Shanghai, China

**Keywords:** sodium-glucose co-transporter-2 inhibitors, satisfaction, adverse events, propensity score matching, anti-diabetic agents, diabetes

## Abstract

**Background:** Specific safety issues with sodium-glucose co-transporter-2 (SGLT2) inhibitors such as infection, fractures, worsening of renal function and euglycemic ketoacidosis have been raised. Concerns about adverse events might limit the use of this drug class. The satisfaction with SGLT2 inhibitors treatment in Chinese patients with type 2 diabetes mellitus (T2DM) is unknown.

**Material and Methods:** Patients with T2DM who visited the hospital between October 2019 and June 2020 were included in this retrospective analysis. Patients were divided into SGLT2 inhibitors used group or not. The Satisfaction with Oral Anti-Diabetic Agent Scale (SOADAS) questionnaire and self-reported AEs were obtained at 3 months of follow-up. Propensity score matching (PSM) was performed to adjust for confounding factors. Univariate and multivariable linear regression models were used to explore potential risk factors associated with overall satisfaction.

**Results:** A total of 145 T2DM patients were included, with 76 SGLT2 inhibitors users and 69 non-users. Patients administered with SGLT2 inhibitors presented with increased overall satisfaction (mean [SE]: 22.8 [0.67] vs. 20.6 [0.64], *p* = 0.016) and overall satisfaction rate (n [%]: 40 [52.6%] vs 21 [30.4%], *p* = 0.007) when compared to other anti-diabetic agents. The use of SGLT2 inhibitors significantly improved satisfaction of glycemic control ability (mean [SE]:3.9 [0.12] vs. 3.5 [0.12], *p* = 0.027), diabetic symptom’s control ability (3.5 [0.15] vs. 3.0 [0.15], *p* = 0.027), glycemic control speed (3.9 [0.11] vs. 3.4 [0.12], *p* = 0.011), medication tolerability (3.9 [0.10] vs. 3.5 [0.12], *p* = 0.012), and overall satisfaction (4.0 [0.11] vs. 3.6 [0.12], *p* = 0.037), but it did not improve satisfaction of medication effect on bodyweight (3.8 [0.11] vs. 3.4 [0.11], *p* = 0.166). After adjusting confounding factors (47 patients for each group), consistent results were obtained. No significant differences of self-reported clinical AEs were observed between SGLT2 inhibitors users and non-users. Multivariable regression analyses verified that the use of SGLT2 inhibitors was associated with increased levels of satisfaction.

**Conclusions:** The use of SGLT2 inhibitors was associated with increased levels of satisfaction in T2DM patients, but not associated with overall clinical safety. Self-reported AEs were not related to the satisfaction with the use of anti-diabetic agents.

## Introduction

China has the largest number of patients with type 2 diabetes mellitus (T2DM) in the world, with more than 100 million people with T2DM (Ma et al., 2014). Over recent decades, the prevalence of T2DM in China has increased from 1% in the 1980s, to 5.5% in 2001, to 9.7% in 2008, and 11.6% in 2010 (Ma et al., 2014). T2DM has become a critical health burden worldwide due to its increasing prevalence and related disability and mortality (Li et al., 2020). Untreated or poorly treated T2DM leads to microvascular damage such as retinopathy and nephropathy or macrovascular events such as myocardial infarction (Nauck et al., 2021). These complications are closely associated with individual factors, glycaemic control, and glucose-lowering therapies (Nauck et al., 2021). Many new treatments have emerged over the decade. Metformin remains as the first-line medication for T2DM patients according to American Diabetes Association Guidelines in 2021 (Vijan, 2019; Association, 2021). Other treatment options such as sodium-glucose co-transporter 2 (SGLT2) inhibitors are recommended as the initial treatment for T2DM patients with cardiovascular diseases based on the guidelines of the European Society of Cardiology and the European Association for the study of Diabetes (Cosentino et al., 2020). SGLT2 inhibitors were first introduced in the United States in 2013 (Singh and Kumar, 2018). They control blood glucose levels by reducing renal tubular glucose reabsorption, resulting in the excretion of urine glucose (Wu et al., 2016; Fitchett, 2019; Neuen et al., 2019). Prior studies emphasized the beneficial effects of SGLT2 inhibitors, but their safety issues were ignored. With the extensive use of SGLT2 inhibitors, adverse events (AEs), such as infection-related AEs (Liu et al., 2017; Puckrin et al., 2018) and renal-related AEs (Perlman et al., 2017; Toyama et al., 2019) have been raised. Our previous meta-analysis validated the United States Food and Drug Administration (FDA) safety alerts and found additional safety issues, such as osmotic diuresis-related AEs, volume-related AEs, and hypoglycaemia (Shi et al., 2019). Assessing whether the safety results of these clinical trials apply to the treatment of T2DM patients is important to determine the application of SGLT2 inhibitors. In addition, treatment satisfaction measures are essential for the successful treatment of diabetes, which is beyond the usual efficacy and safety profiles of specific drugs (Chirila et al., 2016). Some patients may not want to use SGLT2 inhibitors due to their potential safety concerns, so it is common to expect worse patient satisfaction with the use of these drugs. Thus, evaluating the satisfaction of SGLT2 inhibitors is essential and urgent.

In diabetes treatment, several satisfaction questionnaire have been developed and validated (Saisho, 2018; Ida et al., 2020). A questionnaire named “The Satisfaction with Oral Anti-Diabetic Agent Scale (SOADAS)”, was developed to measure treatment satisfaction for oral anti-diabetic agents (Donatti et al., 2008). Then it was translated to Chinese (Lin et al., 2018). The test assessed the satisfaction of hypoglycaemic agent therapy. In this study, we will translate and validate the SOADAS, and it was applied for the satisfaction surveys of T2DM patients. Currently, only three studies have evaluated satisfaction with the use of SGLT2 inhibitors in patients with type 2 diabetes mellitus (Chirila et al., 2016; Nakajima et al., 2018) and those in type 1 diabetes mellitus (Ishibashi et al., 2021). Specific gliflozin or single arm studies (Chirila et al., 2016; Nakajima et al., 2018; Ishibashi et al., 2021) failed to identify any individual gliflozin has improvement scores of the satisfaction outcomes as compared with other oral anti-diabetic agents. However, there is limited evidence supporting the effects of SGLT2 inhibitors on satisfaction. In this study, we aim to evaluate the SGLT2 inhibitors’ impact use on patient satisfaction and clinical AEs.

## Materials and Methods

### Study Population

Patients with T2DM admitted to the Department of Endocrinology of Ren Ji Hospital were recruited from October 2019 to June 2020. Patients included the following criteria: 1) diagnosed with T2DM; 2) were willing to complete a satisfaction survey and self-reported AEs report during follow-up (3 months after hospital discharge); 3) treated with more than two types of hypoglycaemic agents (either oral, intravenous, or subcutaneous); and 4) aged 18 years or older. Patients were excluded if they had the following 1) underwent lifestyle interventions, and 2) expressed reluctance to finish the satisfaction survey and follow-up.

### Trial Design

Patients enrolled in this study were divided into two groups based on the presence or absence of SGLT2 inhibitors. One group was sustained the use of SGLT2 inhibitors (dapagliflozin, empagliflozin, or canagliflozin) as hypoglycaemic agents for more than three months, which was defined as the group of prescription of SGLT2 inhibitors. The other group using non-SGLT2 inhibitors, which including metformin, sulfonylureas, alpha glycosidase inhibitors, thiazolidinediones, glinides, dipeptidyl peptidase-4 inhibitors, thiazolidinediones, insulin, glucagon-like peptide-1 receptor agonist, etc., was considered as the group of non-SGLT2 inhibitors hypoglycaemic agents’ therapy.

### Study Endpoints and Satisfaction Assessments

All patients enrolled in this study were treated with hypoglycaemic agents for more than three months. After patients were discharged from the hospital and underwent three months treatment of hypoglycaemic agents, the satisfaction of each patient was assessed. A Chinese translated SOADAS (License number: 5212801457760) was borrowed to assess patients’ satisfaction in terms of being treated with hypoglycaemic agents. This questionnaire included six items. Each item was scored on a 5-point scale anchored based on Likert 5 grade scale at 1 (extremely dissatisfied) to 5 (extremely satisfied) points (Table 1). The SOADAS was used to assess the satisfaction of all kinds of anti-diabetic agents, not only for oral dosage form. To ensure the overall quality of the SOADAS on the non-oral anti-diabetic agent, the internal consistency reliability and validity were analysed. After the questionnaire was generated through the online questionnaire (www.wenjuan.com), the endocrinologist sent it to the patients through WeChat to complete the satisfaction survey.

**TABLE 1:** SOADAS questionnaire.

Item number	EDSF	DSF	G	SF	ESF
Q1: ability to control blood sugar	1	2	3	4	5
Q2: ability to control diabetic symptoms	1	2	3	4	5
Q3: how quickly medication-controlled blood sugar	1	2	3	4	5
Q4: medication’s effect on weight	1	2	3	4	5
Q5: tolerability of the medication	1	2	3	4	5
Q6: overall satisfaction	1	2	3	4	5
Total satisfaction	5 to 30				

Abbreviations: SOADAS, The Satisfaction with Oral Anti-Diabetic Agent Scale; ED, extremely dissatisfaction, D, dissatisfaction, G, general, S, satisfaction, ES, extremely satisfaction. The total score reached 80% of the total points (overall scores ≥24) was defined as satisfaction, and the overall score <24 was defined as unsatisfaction. Besides, for each item, the score ≥4 was defined as satisfaction, and the score = 1, 2 or 3 was defined as unsatisfaction.

### Data Collection and Outcome Measures

Patient characteristics (demographics, diagnosis, and diabetes-related indices) were recorded from the medical charts and hospital electric information systems. Age, sex, bodyweight, body mass index (BMI), combined diseases, and several diabetes-related parameters for patients’ baseline information on admission were collected. In addition, combined diseases and risks, such as hypertension, coronary heart disease, chronic kidney disease, hyperlipidaemia, etc, were collected. For diabetes-related parameters, the diabetic duration, HbA1c, fasting plasma glucose (FPG) and postprandial plasma glucose (PPG) were collected. In addition, the combined drugs information in each patient, especially the details of hypoglycaemic agents were recorded.

Each item and the overall score of SOADAS questionnaire were collected as the primary outcomes. Self-reported AEs were collected as the secondary outcomes. The items of AEs included in this study were based on a previous study that conducted their selection based on whether it was convenient for self-judgment (Shi et al., 2019). All data were collected three months after the patients were discharged from the hospital and remedied with anti-diabetic agents.

### Statistical Analyses

The reliability and validity of the questionnaire were analysed. Cronbach’s alpha value was used to assess the internal consistency of the proposed constructs. A Cronbach’s alpha value above 0.7 showed that the questionnaire was reliable. The questionnaire of SOADAS was classic, which ensured the content validity. The face validity was checked by pharmacists and endocrinologists who had more than five years of experience in T2DM treatment to extend this questionnaire to the satisfaction evaluation of non-oral anti-diabetic agents. Principal component analysis was applied to test the unidimensionality of the scale. Construct validity was examined by exploratory factor analysis of each questionnaire item to identify the structure of the scale. A coefficient of Kaiser-Meyer-Olkin (KMO) measure of sampling adequacy above 0.5 and a *p*-value of Bartlett’s test of sphericity under 0.05 considered that the construct validity of the questionnaire was good.

SOADAS questionnaire that reached 80% or an overall score ≥24 was defined as satisfaction with the anti-diabetic agents’ treatment, and the overall score <24 was defined as unsatisfaction. In addition, for each item, the score ≥4 was defined as satisfaction, and the score of = 1, 2, or 3 was defined as unsatisfaction. Continuous variables were described using mean with standard error (SE), and compared with the unpaired Student’s t-tests or Wilcoxon signed-rank tests between the SGLT2 inhibitors and non-SGLT2 inhibitors groups. Categorical variables were described as numbers and percentages, and they were compared using the chi-square test or Fisher’s exact tests. Propensity score matched (PSM) analysis was used to correct the differences in patient characteristics between SGLT2 inhibitors and non-SGLT2 inhibitors groups (Oh et al., 2016). Briefly, the propensity score was estimated by multiple logistic regression analysis, which included the corrected variables (BMI, numbers of hypoglycemic agents, etc.). The matching tolerance obtained by matching was evaluated by calculating absolute standardized differences in covariates within the two groups. For the measure covariates, the recommended balance value in this study was 0.02. A multivariable linear regression model was used to explore the potential risk factors associated with overall satisfaction. Two criteria were considered necessary to enter a variable in the multivariable analysis model: 1) The *p*-value of the univariate variable of the influence factor with overall satisfaction ≤0.05; and 2) According to previous data, there was a reasonable relationship with the influence factors of T2DM patients’ overall satisfaction. All statistical analyses were evaluated using the SPSS 22.0 software (SPSS Inc., Chicago, Illinois, United States), and *p <* 0.05 was considered significant.

## Results

### Patient Characteristics

**FIGURE 1:**
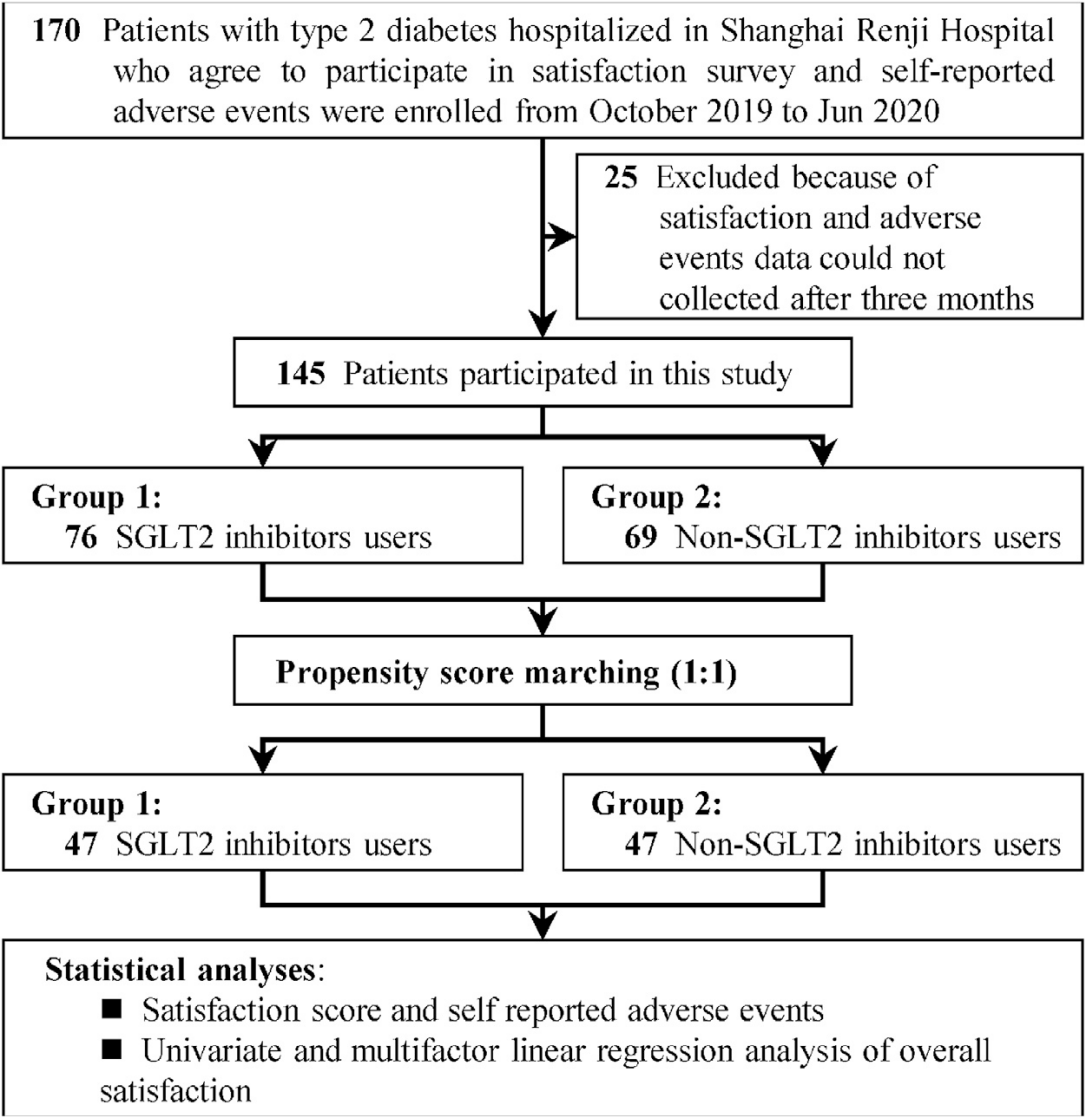
The study follow diagram. Abbreviation: SGLT2: sodium-glucose co-transporter 2.

The flow diagram of this study is presented in Figure 1. A total of 145 patients completed the satisfaction survey and self-evaluated AEs report. Among the 145 patients, 76 used SGLT2 inhibitors, and 69 used other anti-diabetic agents. The demographics and characteristics of T2DM patients enrolled are listed in Table 2. The mean age of patients was 54.3 years, and 73.1% of the patients were male. The mean duration of diabetes was 8.6 years, and the mean HbA1c% was 9.7%. Before PSM, the features of combined disease and risks, diabetes-related parameters, combined drugs were comparable. However, the item of anti-diabetic drugs use showed that the SGLT2 inhibitors group was associated with higher BMI levels (mean [SE]: 26.3 [0.4] vs. 24.2 [0.5]; *p* = 0.003) and more kinds of the anti-diabetic agents use (mean [SE]: 3.1 [0.1] vs. 2.5 [0.1], *p* = 0.001) compared to the non-SGLT2 inhibitors patients. After PSM, the above indicators were well balanced, which resulted in 47 patients for each group. For specific anti-diabetic drugs, patients in SGLT2 inhibitors group associated with less use of alpha-glycosidase inhibitors (n [%]: 21 [44.7] vs. 31 [66]; *p* = 0.038), sulfonylureas (n [%]: 2 [4.3] vs. 8 [17], *p* = 0.045), DPP4 inhibitors (n [%]: 11 [23.4] vs. 24 [51.1]; *p* = 0.006) when compared to non-SGLT2 inhibitors group.

**TABLE 2:** Demographics and characteristics of patients.

Characteristics	Total (*n* = 145)	Before PSM		After PSM	
		NSGLT2i (*n* = 69)	SGLT2i (*n* = 76)	NSGLT2i (*n* = 47)	SGLT2i (*n* = 47)
Patients’ characteristics					
Age (year)	54.3 ± 1.2	54.9 ± 1.8	53.7 ± 1.6	56.0 ± 2.1	55.6 ± 2.1
Male	106 (73.1)	46 (66.7)	60 (78.9)	31 (66.0)	36 (76.6)
Body weight (kg)	73.2 ± 1.3	68.1 ± 1.8	78.0 ± 1.8^**^	71.1 ± 2.2	74.8 ± 2.2
Body mass index	25.3 ± 0.4	24.2 ± 0.5	26.3 ± 0.4^**^	25.2 ± 0.7	25.4 ± 0.5
Combined disease and risks					
Overall (n)	7.6 ± 0.3	7.6 ± 0.4	7.5 ± 0.3	8.0 ± 0.4	7.3 ± 0.4
Hypertension	71 (49.0)	32 (46.4)	39 (51.3)	25 (53.2)	25 (53.2)
Coronary heart disease	15 (10.3)	6 (8.7)	9 (11.8)	5 (10.6)	4 (8.5)
Chronic kidney disease	12 (8.3)	6 (8.7)	6 (7.9)	5 (10.6)	4 (8.5)
Hyperlipemia	68 (46.9)	27 (39.1)	41 (53.9)	18 (38.3)	25 (53.2)
eGFR	99.4 ± 1.8	99.6 ± 2.6	99.3 ± 2.4	97.8 ± 3.5	97.6 ± 3.2
Diabetes related indicators					
Diabetic duration (year)	8.6 ± 0.7	9.5 ± 1.1	7.9 ± 0.9	10.3 ± 1.3	8.0 ± 1.1
HbA1c%	9.7 ± 0.2	9.6 ± 0.3	9.9 ± 0.3	9.8 ± 0.4	9.6 ± 0.3
FPG (mmol/L)	7.6 ± 0.2	7.1 ± 0.3	7.9 ± 0.3	7.4 ± 0.3	7.2 ± 0.3
PPG (mmol/L)	14.3 ± 0.4	14.5 ± 0.6	14.1 ± 0.5	14.1 ± 0.7	14.5 ± 0.6
Combined drugs use					
Overall (n)	6.0 ± 0.2	5.7 ± 0.3	6.2 ± 0.3	6.4 ± 0.3	5.9 ± 0.3
Antihypertensive drugs	51 (35.2)	26 (37.7)	25 (32.9)	20 (42.6)	16 (34.0)
Lipid-lowering drugs	103 (71.0)	47 (68.1)	56 (73.7)	30 (63.8)	30 (63.8)
Antiplatelet drug	41 (28.3)	15 (21.7)	26 (34.2)	12 (25.5)	16 (34.0)
UA lowering drugs	31 (21.4)	11 (15.9)	20 (26.3)	8 (17.0)	11 (23.4)
Anti-diabetic drugs use					
Overall (n)	2.8 ± 0.1	2.5 ± 0.1	3.1 ± 0.1**	2.9 ± 0.1	2.9 ± 0.1
Metformin	107 (73.8)	45 (65.2)	62 (81.6)*	37 (78.7)	34 (72.3)
α glycosidase inhibitor	75 (51.7)	37 (53.6)	38 (50.0)	31 (66.0)	21 (44.7)*
Sulfonylureas	19 (13.1)	10 (14.5)	9 (11.8)	8 (17.0)	2 (4.3)*
non-Sulfonylurea	8 (5.5)	5 (7.2)	3 (3.9)	5 (10.6)	3 (6.4)
DPP4i	48 (33.1)	31 (44.9)	17 (22.4)**	24 (51.1)	11 (23.4)**
GLP-1 RAs	20 (13.8)	9 (13.0)	11 (14.5)	9 (19.2)	4 (8.5)
SGLT2i	76 (52.4)	-	76 (100)	-	47 (100)
Insulin	50 (34.5)	31 (44.9)	19 (25.0)**	18 (38.3)	12 (25.5)

Results were presented with mean ± SE., Abbreviations; PSM, propensity score matching, SGLT2i, sodium-glucose co-transporter 2 inhibitors, NSGLT2i, non- SGLT2i, DPP4i, dipeptidyl peptidase IV, inhibitors; GLP-1, ras, Glucagon like peptide-1 receptor agonists. *p < 0.05 and **p < 0.01 when compared to NSGLT2i group.

### Reliability and Validity of Questionnaire

The Cronbach’s *α* value of this questionnaire was 0.95, which validated the good internal consistency of the tool. Principle component analysis represented that the questionnaire used in this study was unidimensional. The first factor accounted for 81.4% of the total variance, and the eigenvalue was 4.89, which was significantly higher than the second component (eigenvalue = 0.49) and the following elements. The average score of a separate item ranged from 3.3 to 3.8, while the average total score was 21.8. Few patients were “extremely dissatisfied” with any item (0–1.38%), while 25.5–31.7% of the participants answered “extremely satisfied” with a single item. Overall, 28 (19.3%) of the participants reached the highest possible score (total score = 30), indicating a high level of satisfaction with their current medications. All six items had an item-total correlation value above 0.9 (Table 3).

**TABLE 3:** Characteristics of the questionnaire used in this study (*n* = 145).

Item	Mean ± SE	Score range	Floor (%)	Ceiling (%)	Item total correlation (r)*
Q1	3.7 ± 0.08	2–5	0	29.7	0.933
Q2	3.3 ± 0.11	2–5	0	27.6	0.941
Q3	3.7 ± 0.08	1–5	0.69	26.9	0.933
Q4	3.6 ± 0.08	1–5	1.38	25.5	0.960
Q5	3.7 ± 0.08	2–5	0	26.2	0.938
Q6	3.8 ± 0.08	2–5	0	31.7	0.938
Total	21.8 ± 0.47	12–30			
Mean	3.6 ± 0.08	2–5			

Questions: Q1: ability to control blood sugar; Q2: ability to control diabetic symptoms; Q3: how quickly medication-controlled blood sugar; Q4: medication’s effect on weight; Q5: tolerability of the medication; Q6: overall satisfaction. Floor means the score of this item is 0, while ceiling means the score of this item is 5.

### Comparison of Satisfaction Scores

**FIGURE 2:**
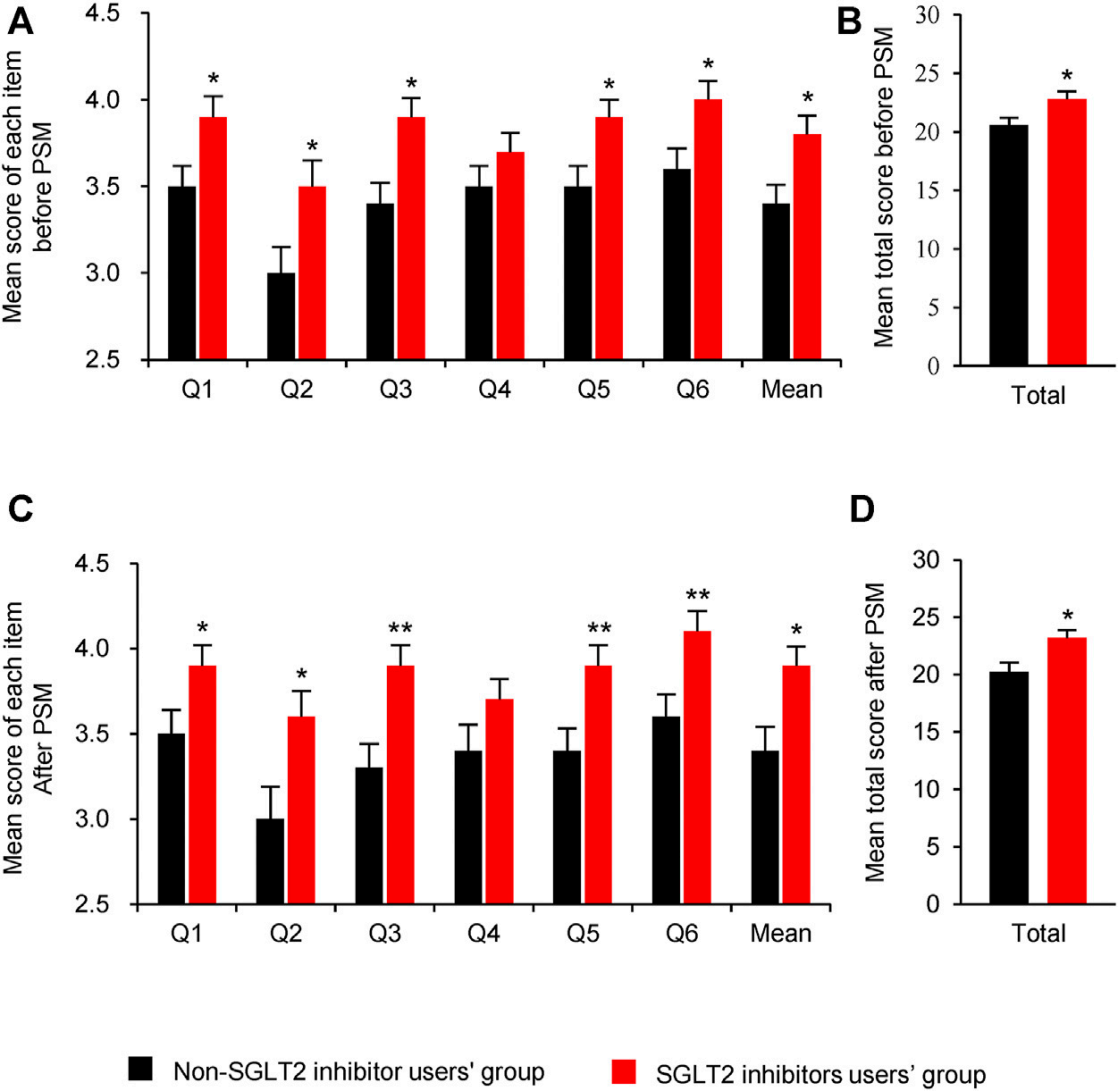
Comparison of satisfaction scores between SGLT2 inhibitors users and non-SGLT2 inhibitors users. **(A)**: Mean score of each item before PSM; **(B)**: Mean total score before PSM; **(C)**: Mean score of each item after PSM; **(D)**:Mean total score after PSM. Questions: Q1: ability to control blood sugar; Q2: ability to control diabetic symptoms; Q3: how quickly medication-controlled blood sugar; Q4: medication’s effect on weight; Q5: tolerability of the medication; Q6: overall satisfaction. Abbreviations: PSM, Propensity score matching; SGLT2i: sodium-glucose co-transporter 2 inhibitors. Data were represented as Mean ± SE. * P < 0.05 and ** P < 0.01 vs. non-SGLT2 inhibitor users’ group.

Before PSM, the use of SGLT2 inhibitors significantly improved satisfaction of glycaemic control ability (Q1, mean [SE]: 3.9 [0.12] vs. 3.5 [0.12], *p* = 0.027), diabetic symptoms control ability (Q2, mean [SE]: 3.5 [0.15] vs. 3.0 [0.15], *p* = 0.027), speed of medication control ability (Q3, mean [SE]: 3.9 [0.11] vs.3.4 [0.12], *p* = 0.011), tolerability of the medication (Q5, mean [SE]: 3.9 [0.10] vs.3.5 [0.12], *p* = 0.012) and overall satisfaction (Q6, mean [SE]: 4.0 [0.11] vs.3.6 [0.12], *p* = 0.037); however, medication effect on bodyweight was not significant (Q4, mean [SE]: 3.8 [0.11] vs.3.4 [0.11], *p* = 0.166) (Figure 2A). Patients administered with SGLT2 inhibitors presented with increased overall satisfaction score of glycaemic control ability (mean [SE]: 22.8 [0.67] vs. 20.6 [0.64], *p* = 0.016) compared to non-SGLT2 inhibitors group (Figure 2B).

After PSM, all the items were consistent with the primary outcomes before PSM (Figures 2C,D). But only three items, which were the speed of medication control ability (Q3), tolerability of the medication (Q5) and overall satisfaction (Q6) were significant after PSM.

### Comparison of Satisfaction Rate

The results of each item and total satisfaction rate before and after PSM are summarized in Table 4. Before PSM, the total satisfaction rate was significant higher in SGLT2 inhibitors group than non-SGLT2 inhibitor group (52.6 vs. 30.4%, *p* = 0.005). After PSM, the ratio gap of total satisfaction widened between SGLT2 inhibitors group and non-SGLT2 inhibitor group (53.2 vs. 23.4%, *p* = 0.003). After PSM, SGLT2 inhibitors-based treatment resulted in more satisfaction of diabetic symptoms (Q2, *p* = 0.031), blood glucose control speed (Q3, *p* = 0.006), tolerability of the medication (Q5, *p* = 0.031), and overall satisfaction (Q6, *p* = 0.030) than non-SGLT2 inhibitor therapy.

**TABLE 4:** Satisfaction rate of each item before and after PSM.

Item	Before PSM		After PSM	
	Non-SGLT2i (*n* = 69)	SGLT2i (*n* = 76)	Non-SGLT2i (*n* = 47)	SGLT2i (*n* = 47)
Q1	32 (46.4)	46 (60.5)	21 (44.7)	29 (61.7)
Q2	29 (42.0)	45 (59.2)*	19 (40.4)	29 (61.7)*
Q3	27 (39.1)	45 (59.2)^*^	15 (31.9)	28 (59.6)**
Q4	28 (40.6)	40 (52.6)	17 (36.2)	25 (53.2)
Q5	29 (42.0)	44 (57.9)	18 (38.3)	28 (59.6)*
Q6	34 (49.3)	47 (61.8)	22 (46.8)	32 (68.1)*
Total	21 (30.4)	40 (52.6)**	11 (23.4)	25 (53.2)**

Questions: Q1: ability to control blood sugar; Q2: ability to control diabetic symptoms; Q3: how quickly medication-controlled blood sugar; Q4: medication’s effect on weight; Q5: tolerability of the medication; Q6: overall satisfaction. Definition: Satisfaction, the score of Q1∼Q6 ≥ 4 or overall scores ≥24; unsatisfaction, the score of Q1∼Q6 < 4 or overall score <24. Results were presented as number of satisfied and percentage. SGLT2i vs Non-SGLT2i, *p < 0.05 and **p < 0.01.; Abbreviations: PSM, propensity score matching, SGLT2i, sodium-glucose co-transporter 2 inhibitors.

### Self-Reported Adverse Events

The self-reported AEs for 3 months follow-up are presented in Table 5. The incidence of AEs was 40.8% (31/76) in the SGLT2 inhibitors group, while it was 37.7% (26/69) in the non-SGLT2 inhibitor group. SGLT2 inhibitors application did not statistically increase any AEs rate when compared with non-users of SGLT2 inhibitors (Table 5).

**TABLE 5:** Adverse events.

Adverse events	All patients (*n* = 145)	Non-SGLT2i (*n* = 69)	SGLT2i (*n* = 76)	*p* Value
Adverse events (No. patients)	57 (39.3)	26 (37.7)	31 (40.8)	0.416
Any adverse events (No. events)	1.2 ± 0.2	1.1 ± 0.2	1.3 ± 0.3	0.516
Infections and infestations	11 (7.6)	5 (7.3)	6 (7.9)	0.567
Urinary tract infection	9 (6.2)	4 (5.8)	5 (6.6)	0.561
Genital mycotic infection	1 (0.7)	1 (1.5)	0 (0)	0.476
Nasopharyngitis	1 (0.7)	0 (0)	1 (1.3)	0.524
Bronchitis	1 (0.7)	1 (1.5)	0 (0)	0.476
Gastrointestinal disorders	76 (52.4)	36 (52.2)	40 (52.6)	0.475
Nausea	4 (2.8)	3 (4.4)	1 (1.3)	0.274
Vomit	3 (2.1)	2 (2.9)	1 (1.3)	0.464
Constipation	10 (6.9)	4 (5.8)	6 (7.9)	0.435
Stomachache	2 (1.4)	0 (0)	2 (2.6)	0.273
Diarrhea	7 (4.8)	1 (1.5)	6 (7.9)	0.075
Stomach upset or indigestion	11 (7.6)	4 (5.8)	7 (9.2)	0.325
Loss of appetite	7 (4.8)	3 (4.4)	4 (5.3)	0.554
Osmotic diuresis or volume related AEs	10 (6.9)	3 (4.4)	7 (9.2)	0.206
Increased water drinking	9 (6.2)	3 (4.4)	6 (7.9)	0.297
Increased urine	6 (4.1)	3 (4.4)	3 (4.0)	0.613
Postural hypotension	2 (1.4)	0 (0)	2 (2.6)	0.273
Musculoskeletal disorders	13 (9.0)	6 (8.7)	7 (9.2)	0.574
Arthralgia	6 (4.1)	4 (5.8)	2 (2.6)	0.296
Back pain	4 (2.8)	2 (2.9)	2 (2.6)	0.653
Limb pain	3 (2.1)	0 (0)	3 (4.0)	0.141
Hypoglycemia	16 (11.0)	9 (13.0)	7 (9.2)	0.319
Asymptomatic hypoglycemia	1 (0.7)	0 (0)	1 (1.3)	0.524
Symptomatic hypoglycemia	15 (10.3)	9 (13.0)	6 (7.9)	0.229
Other adverse events				
Dizzy	4 (2.8)	2 (2.9)	2 (2.6)	0.653
Headache	1 (0.7)	1 (1.5)	0 (0)	0.476
Edema	2 (1.4)	0 (0)	2 (2.6)	0.273
Rash	8 (5.5)	2 (2.9)	6 (7.9)	0.171
Fractures	1 (0.7)	1 (1.5)	0 (0)	0.476

Abbreviations: SGLT2i: sodium-glucose co-transporter 2 inhibitors; AEs: adverse events; Urinary tract infection and genital mycotic infection were considered possible by patients themselves; Symptomatic hypoglycemia mainly included sweating, dizzy, patients needed to eat something to recover. *p* value referred to SGLT2 inhibitors versus non-SGLT2, inhibitors.

### Risk Factors Associated With Overall Satisfaction

Univariate linear regression analysis identified several factors that were statistically associated with overall satisfaction. Factors such as age, combined diseases, diabetic duration, combined drugs, and the number of anti-diabetic agents negatively correlated with overall satisfaction. In contrast, bodyweight, BMI, and uric acid were positively related. For anti-diabetic agents, SGLT2 inhibitors increased the scores of overall satisfactions (*p* < 0.05, Supplementary Table S1), whereas *α*-glycosidase inhibitors, sulfonylureas, DPP4 inhibitors, and insulin decreased the scores of overall satisfactions (*p* < 0.05). Additionally, self-reported AEs (β coefficient = 0.490; *p* = 0.167) were not related to the satisfaction of anti-diabetic agents (Supplementary Table S1). Notably, only numbers of anti-diabetic agents used were negatively and SGLT2 inhibitors positively correlated with overall satisfaction in the multifactor linear regression analysis (*p* < 0.05 for each variable) (Supplementary Table S2).

## Discussion

Oral anti-diabetic agents are not the only option for diabetes treatment. Due to numerous new treatments and medications come out one after another, T2DM **t**reatment guidelines are rapidly updated over the past 10 years (Wilkinson et al., 2018; Bang et al., 2020). Following the newest American Diabetes Association ADA guideline, the early introduction of insulin should be considered if symptoms of hyperglycaemia are present or when HbA1c levels or blood glucose level are very high (Association, 2021). Since T2DM is a major factor for cardiovascular disease, the presence of comorbidities such as heart failure and left ventricular dysfunction should be considered in diabetes treatment (Newman et al., 2018). In recent years, two classes of new anti-diabetic agents, SGLT2 inhibitors and glucagon-like peptide-1 (GLP-1) receptor agonists shed light on cardiovascular and renal benefits in patients with established or high risks of cardiovascular disease (Dave et al., 2020). These two new anti-diabetic medications are widely used in the clinics, but there has been no satisfaction survey of Chinese patients with T2DM on the use of SGLT2 inhibitors. The SOADAS questionnaire is the first treatment satisfaction instrument specific to oral anti-diabetic agents (Donatti et al., 2008). However, assessment of satisfaction of injective anti-diabetic agents was also necessary because the included hospitalized patients had high levels of HbA1c and blood glucose, so insulin use was inevitable. To make the tool applicable to oral and injective antidiabetic agents, we invited a group of health care professionals to evaluate the SOADAS used in not only oral anti-diabetic agents, but also in injection anti-diabetic agents. The SOADAS questionnaire was verified as a reliable measurement for assessing patients’ satisfaction in both oral and injective anti-diabetic agents, with high internal consistency. Thus, in this study, we evaluated the impact of SGLT2 inhibitors on patients’ satisfaction and AEs using the SOADAS questionnaire.

Currently, only 3 studies have assessed the satisfaction associated with SGLT2 inhibitors use. A Japanese patient-reported outcome (PRO) study that included 221 T2DM patients found a significantly improved satisfaction of treatment with dapagliflozin (Nakajima et al., 2018). Ryoichi Ishibashi et al. (Ishibashi et al., 2021) concluded that initiation use of ipragliflozin could improve glycaemic indexes and average satisfaction scores in 24 patients with Type 1 diabetes. Both the above studies were single-arm studies without reducing the influence of other oral anti-diabetic agents. Results from the early empagliflozin satisfaction study also failed to detect a significant difference in satisfaction scores between empagliflozin and glimepiride. Although, it lowered the perceived frequency of hyperglycemia and hypoglycemia from weeks 28 onward (Chirila et al., 2016). Our study found that the use of SGLT2 inhibitors could increase the scores of glycaemic control ability, diabetic symptom’s ability, speed of medication control ability, tolerability of the medication, and overall satisfaction except for bodyweight control. The explanations of improvement in patient satisfaction for T2DM are multifactorial, in which the improvement in heart failure symptoms can mainly drive the results. A multimodality study about empagliflozin affects diastolic function in heart failure with reduced ejection fraction (HFrEF) found it could ameliorate diastolic dysfunction and left ventricular fibrosis (Santos-Gallego et al., 2021a). Another secondary analysis of patients enrolled in the EMPA-TROPISM study also improved adiposity, interstitial myocardial fibrosis, and aortic stiffness (Requena-Ibáñez et al., 2021). This may explain the possible underline reasons SGLT2 inhibitors could improve quality of life and patient satisfaction in both HFrEF (Santos-Gallego et al., 2021b) and HFpEF patients (Nassif et al., 2021). The inconsistent results related to the satisfaction on bodyweight control may be partly attributed to the higher baseline bodyweight and BMI in the SGLT2 inhibitors users compared to non-SGLT2 inhibitors users. To further verify these findings and reduce confounding factors in this study, the PSM method was used to control factors such as BMI and the number of anti-diabetic drugs used. The adjusted results of PSM were consistent with the original results, and there were statistically significant differences. Several underlined reasons might explain the phenomena of patient satisfaction on weight control abilities with different treatment strategies. First, many participants had difficulty in answering the item examining the anti-diabetic drug’s effect on weight. The patients tended to attribute weight change to their diet or exercise rather than the drug’s effect (Lin et al., 2018). Second, most clinical trials showed the positive impact of SGLT2 inhibitors on weight loss. However, real-world evidence and clinical experience showed significant heterogeneity in the magnitudes of the actual weight loss, which is significantly less than anticipated (Brown et al., 2019). Nevertheless, weight loss improves glycaemic control and weight-related comorbidities, so physicians or patients prefer drugs that may reduce bodyweight.

Certain safety issues, such as infection-related (Liu et al., 2017), renal-related AEs (Menne et al., 2019), and diabetic ketoacidosis (Liu et al., 2020), have been raised with the extensive clinical application of SGLT2 inhibitors. We also found medication with SGLT2 inhibitors was connected with a higher risk of infections, osmotic diuresis-related AEs, volume-related AEs, renal-related AEs, and hypoglycaemia when randomized clinical trials evidence were pooled (Shi et al., 2019). However, the current study showed that SGLT2 inhibitors did not cause more overall safety issues than non-SGLT2 inhibitors on 3 months follow-up. Our previous meta-analysis reported that the incidence of any AEs was 66.9% (19258/28803) in the SGLT2 inhibitors group, while it was 68.5% (9648/14091) in the placebo group, indicating SGLT2 inhibitors did not increase the risks of any AEs (Shi et al., 2019). The secondary outcome of our study was the self-reported AEs. The incidence of any AEs was lower than the results from our previous meta-analysis (Shi et al., 2019), wherein the SGLT2 inhibitors group was 40.8% (31/76), and in the non-SGLT2 inhibitors group was 37.7% (26/69). The following reasons may explain the differences in lower incidence in this observational study. First, we used self-reported AEs. Whether the patient lacked the cognitive ability, and the definition of reproductive system infection is unclear, so only one patient reported genital pruritus. Second, the participants’ diseases, comorbidity, and combined drugs were different, making a distinction between this study and randomized clinical trials (RCTs). Finally, the follow-up durations were relatively shorter than many RCTs.

Overall, the strengths of this study are related to the evaluation of SGLT2 inhibitors’ effects on satisfaction and self-reported AEs in T2DM. The validated SOASAS could be used as an outcome measure in clinical practice of anti-hyperglycaemic agents, not limited to oral anti-diabetic drugs. The PSM analysis was also a strength in this study. Given that the baseline characteristics between the SGLT2 inhibitors and other anti-diabetic medication groups were not comparable, the PSM method was used to reduce the possibility of confounding factors.

Several limitations in this study still needed to be considered. First, this study totally included a relatively small number of participants (145 patients, 76 used SGLT2 inhibitors and 69 treated with other anti-diabetic agents). Therefore, a larger sample size study is needed in the future research. Second, since the participants are mainly patients discharged from our institution, this study may not be applicable to all T2DM patients. However, our study represents relatively severe T2DM patients who need to be hospitalized with poor blood glucose control. Third, the questionnaire was only performed after three months of intervention. A repeated survey should be launched in further research. Fourth, the dose and adherence of anti-hyperglycaemic drugs were not involved in this study, which may influence the results. Finally, self-reported AEs may cause information loss.

## Conclusion

SGLT2 inhibitors, compared to non-SGLT2 inhibitors, were associated with higher satisfaction scores and rates in terms of blood glucose control ability, diabetic symptoms control ability, blood glucose control speed, medication tolerability, and overall satisfaction. The short-term use of SGLT2 inhibitors were not associated with higher prevalence of self-reported clinical AEs than other anti-diabetic therapies. The multivariable analysis further demonstrated that the use of SGLT2 inhibitors was the positive factor for overall satisfaction. Therefore, based on short-duration data analysis, this study may relieve concerns about the effect of SGLT2 inhibitors use on patients’ satisfaction and clinical AEs.

## Data Availability

The original contributions presented in the study are included in the article/Supplementary Material, further inquiries can be directed to the corresponding authors.

## Supplementary Material

Supplementary Table S1 Univariate linear regression analysis of overall satisfaction

Supplementary Table S2 Multifactor linear regression analysis of overall satisfaction
