# Supplementary material for: Sodium-Glucose Co-Transporter 2 Inhibitors Use Improves the Satisfaction With Anti-diabetic Agent Treatment: A Questionnaire-based Propensity Score-matched Study
Source: Front Pharmacol. 2022 Feb 1;12:787704. doi: 10.3389/fphar.2021.787704 (PMC8844021; doi:10.3389/fphar.2021.787704)
Supplement: Supplementary file 1 [file Table1.docx]

**Table S1∣**Univariate linear regression analysis of overall satisfaction

| Risk Factors | β | β(standardization) | 95%CI | *P* value |
| --- | --- | --- | --- | --- |
| Patients’ characteristics | | | | |
| Age | -0.128 | -0.328 | -0.189 to -0.067 | **<0.001** |
| Body weight | 0.155 | 0.433 | 0.101 to 0.208 | **<0.001** |
| Body mass index | 0.420 | 0.315 | 0.211 to 0.629 | **<0.001** |
| Combined disease and risks | | | | |
| Combined disease (n) | -0.516 | -0.275 | -0.814 to -0.217 | **0.001** |
| Hypertension | 0.225 | 0.020 | -1.642 to 2.091 | 0.812 |
| Coronary heart disease | -2.259 | -0.122 | -5.301 to 0.783 | 0.144 |
| Chronic kidney disease | -0.009 | 0.000 | -3.397 to 3.378 | 0.996 |
| Hyperlipemia | 1.341 | 0.118 | -0.516 to 3.198 | 0.156 |
| eGFR | 0.024 | 0.090 | -0.020 to 0.068 | 0.282 |
| Diabetes | | | | |
| Diabetic duration | -0.271 | -0.392 | -0.377 to -0.165 | **<0.001** |
| HbA1c | 1.500 | 0.095 | -1.095 to 4.095 | 0.255 |
| FPG | -0.168 | -0.077 | -0.529 to 0.193 | 0.358 |
| PPG | -0.352 | -0.250 | -0.619 to -0.084 | **0.011** |
| Combined drugs | | | | |
| Combined drugs (n) | -0.500 | -0.216 | -0.875 to -0.126 | **0.009** |
| Antihypertensive drugs | -0.626 | -0.053 | -2.578 to 1.326 | 0.527 |
| Lipid-lowering drugs | -2.317 | -0.186 | -4.339 to -0.296 | **0.025** |
| Antiplatelet drug | -2.146 | -0.171 | -4.188 to -0.104 | **0.040** |
| UA lowering drugs | 3.918 | 0.284 | 1.735 to 6.100 | **0.001** |
| Hypoglycemic drugs | | | | |
| Hypoglycemic agent (n) | -1.568 | -0.310 | -2.361 to -0.774 | **<0.001** |
| Metformin | -0.898 | -0.070 | -3.015 to 1.219 | 0.403 |
| Alpha glycosidase inhibitor | -2.096 | -0.186 | -3.932 to -0.261 | **0.025** |
| Sulfonylureas | -5.113 | -0.306 | -7.746 to -2.479 | **<0.001** |
| non-Sulfonylurea | 3.563 | 0.144 | -0.482 to 7.608 | 0.084 |
| DPP4i | -2.629 | -0.219 | -4.564 to -0.694 | **0.008** |
| GLP-1 RAs | -0.242 | -0.015 | -2.948 to 2.464 | 0.860 |
| SGLT2i | 2.249 | 0.199 | 0.418 to 4.081 | **0.016** |
| Insulin | -2.196 | -0.185 | -4.126 to -0.266 | **0.026** |
| Drugs effects | | | | |
| Self-reported AEs (n) | 0.490 | 0.115 | -0.208 to 1.188 | 0.167 |

CI: confidence interval; eGFR: estimated glomerular filtration rate; HbA1c: glycosylated hemoglobin; FPG: fasting plasma glucose; PPG: postprandial plasma glucose; UA: uric acid; SGLT2i: sodium-glucose co-transporter 2 inhibitors; DPP4i: dipeptidyl peptidase IV inhibitors; GLP-1 RAs: Glucagon like peptide-1 receptor agonists.
