# Supplementary material for: Sodium-Glucose Co-Transporter 2 Inhibitors Use Improves the Satisfaction With Anti-diabetic Agent Treatment: A Questionnaire-based Propensity Score-matched Study
Source: Front Pharmacol. 2022 Feb 1;12:787704. doi: 10.3389/fphar.2021.787704 (PMC8844021; doi:10.3389/fphar.2021.787704)
Supplement: Supplementary file 2 [file Table2.docx]

**Table S2∣**Multifactor linear regression analysis of overall satisfaction

| Risk Factors | β | β (standardization) | 95%CI | *P* value |
| --- | --- | --- | --- | --- |
| Patients’ characteristics | | | | |
| Age | -0.034 | -0.094 | -0.112 to 0.044 | 0.387 |
| Body weight | 0.017 | 0.048 | -0.109 to 0.143 | 0.791 |
| Body mass index | -0.036 | -0.027 | -0.470 to 0.398 | 0.869 |
| Combined disease and risks | | | | |
| Combined disease (n) | 0.033 | 0.018 | -0.383 to 0.449 | 0.875 |
| Diabetes | | | | |
| Diabetic duration | -0.068 | -0.097 | -0.201 to 0.066 | 0.316 |
| PPG | -0.104 | -0.074 | -0.329 to 0.120 | 0.357 |
| Combined drugs | | | | |
| Combined drugs (n) | 0.404 | 0.162 | -0.222 to 1.030 | 0.203 |
| Lipid-lowering drugs | -0.601 | -0.047 | -2.736 to 1.535 | 0.577 |
| Antiplatelet drug | -0.238 | -0.020 | -2.302 to 1.826 | 0.819 |
| UA lowering drugs | 0.401 | 0.027 | -2.498 to 3.299 | 0.784 |
| Hypoglycemic drugs | | | | |
| Hypoglycemic agent (n) | -1.805 | -0.364 | -3.241 to -0.369 | **0.014** |
| Alpha glycosidase inhibitor | 0.680 | 0.061 | -1.666 to 3.026 | 0.566 |
| Sulfonylureas | -0.963 | -0.061 | -4.136 to 2.210 | 0.548 |
| DPP4i | 1.601 | 0.139 | -0.791 to 3.993 | 0.187 |
| SGLT2i | 2.438 | 0.217 | 0.211 to 4.665 | **0.032** |
| Insulin | -0.353 | -0.029 | -2.906 to 2.201 | 0.784 |

CI: confidence interval; PPG: postprandial plasma glucose; UA: uric acid; SGLT2i: sodium-glucose co-transporter 2 inhibitors; DPP4i: dipeptidyl peptidase IV inhibitors.
